# Latest Experiments with the Virus of Hydrophobia

**Published:** 1881-04

**Authors:** 


### Original Translations.

LATEST EXPERIMENTS WITH THE VIRUS OF HYDROPHOBIA. By BOULEY and PASTEUR.

Bouley says: I have often seen rabid rabbits live one, two, three, even four days. Sheep have become rabid after a period of incubation varying from seventeen to forty days. In rabbits, the usual length of the incubation is from twelve to eighteen days. It is reciprocally contagious by inoculation between the dog, the sheep, and the rabbit. These are my conclusions:

1. The virus of rabies exists only in the saliva, and in the mucous secretion of the buccal cavity; 2. The virus has proven effective after as long as ten days; 3. Under cultivation, the fluids used become crowded with bacteria, and the product of a third, fourth, or fifth cultivation (generation) has caused death in guinea-pigs, in eight, twelve, and twenty-two days. But hydrophobia could not be communicated from these to young dogs; 4. Rabies has been communicated by injecting hypodermically the diseased saliva into the pleural cavity, or into the sciatic nerve. Its ingestion, and its injection into the jugular vein, have been ineffectual; 5. Amputating the part inoculated, half an hour or more after the operation does not prevent the disease; 6. Fowls seem to enjoy an immunity from hydrophobia; 7. Injection of 3 or 4 cc. of diseased saliva into the cellular tissue has given rise to local troubles, and caused a quick septicæmia (?) which killed the dogs in four, five, or eight days; 8. In one case, rabies took place after a year's incubation, in a bitch which had been isolated.

Pasteur thus relates his discovery of a new disease allied to hydrophobia: A child died with hydrophobia December 11, 1880. Four hours after death, I scraped off some buccal mucus with a brush, diluted it with water, and inoculated it immediately in two rabbits. These died about thirty-six hours after inoculation. Other rabbits were inoculated with the saliva, others with the blood, of the first rabbits. Death was still more rapid. A great many inoculations with the saliva, or with the blood of the diseased animals on other rabbits gave similar results. Inoculation with fresh blood caused death generally within the twenty-four hours. At the autopsy, the same lesions were constant in all of the cases. At the seat of punctures, on the abdomen, the venous system is rendered prominent. If death has been delayed, the cellular tissue in the neighborhood is injected with pus, and a new tissue, hard and purulent, forms adhesions between the skin and the abdominal muscles. A point worthy of remark is the swelling of the ganglia (lymphatics) on either side of the trachea, in the groin, and in the axilla, and their hæmorrhagic condition.

The cellular tissue in the groin, in the axilla, and at the points of inoculation, is generally œdematous. The lungs are frequently filled with apoplectic spots. A more constant result is the redness, congestion, and hæmorrhages in the smaller vessels of the trachea. The blood is more fluid, less liable to coagulate is black, viscous, and resembles that of anthrax. As to the symptoms, anorexia promptly appears, coming as early as five or six hours after inoculation. Paralysis sets in, and the animal dies with asphyxia. The mouth is at times frothy after life has ceased. Whether death be the result of inoculation with the saliva, or the blood, the blood of the diseased animal is crowded with bacteria having highly interesting properties. Their shape is that of short cylinders, depressed at the middle, resembling somewhat a figure of 8, with a medium diameter not exceeding one-thousandth of a millimeter, and are surrounded with an areola of doubtful nature.

Experiments in cultivation have proven that the virulence exists in solutions of these organisms, after all foreign substances which might have adhered to the latter have been eliminated. These bacteria must no doubt be the true and only cause of the new disease. The blood of the dead child did not start any growth of bacteria in the solutions in which it was tried. The bacteria in the solution are larger and hang together in the form of beads, but their shape does not differ from that of many other organisms. The solutions act like the blood, or the saliva, after they have been inoculated.

This is a new disease, caused by the presence of a new microscopical parasite, never investigated before this day, and this discovery is another achievement in the etiology of communicable diseases. The most remarkable peculiarity of the new virus is the following: Every one knows how near-related the guinea-pig is to the rabbit, as to anatomical structure, mode of living, and the facility with which it is susceptible of all the same diseases; but the inoculation with infected saliva has given rise to no serious symptoms in that animal as yet; but as only a few weeks have elapsed, they may yet appear.

The new disease differs from rabies in that it has no incubation as the latter. A valuable work by Prof. Galtier, of the Veterinary School of Lyon, states that: 1. Rabies communicated from dog to rabbit does not give rise to symptoms before from four to forty days after inoculation; 2. The dead rabbit presents no anatomical lesions resembling those above enumerated; 3. The blood from dead rabbits cannot communicate the disease.

I have inoculated the new disease in dogs which immediately became very sick, and most of them died in a few days, without the usual symptoms of rabies in the canine species. But why is not this new disease a form of rabies in dogs and rabbits, from humanized virus?

In order to prevent any error, we inoculated in rabbits, without any result, the saliva of asphyxiated rabbits, and that of human bodies dead of common diseases. In case hydrophobia could be referred to a microscopical organism, it would not exceed perhaps the present resources of science to discover means of attenuating the virus, and inoculating it, to impart immunity from hydrophobia, in men as well as in dogs.

The new disease has no connection with septicæmia, which, as we know, readily takes on guinea-pigs.

